# Long-Term Single-Dose Efficacy of a Vesicular Stomatitis Virus-Based Andes Virus Vaccine in Syrian Hamsters

**DOI:** 10.3390/v6020516

**Published:** 2014-01-31

**Authors:** Joseph Prescott, Blair L. DeBuysscher, Kyle S. Brown, Heinz Feldmann

**Affiliations:** 1 Laboratory of Virology, Division of Intramural Research, National Institute of Allergy and Infectious Diseases, National Institutes of Health, Rocky Mountain Laboratories, Hamilton, MT, 59840, USA; E-Mails: prescottjb@niaid.nih.gov (J.P.); debuysscherb@niaid.nih.gov (B.L.D.); 2 Division of Biological Sciences, University of Montana, Missoula, MT, 59840, USA; 3 Vaccine and Infectious Disease Organization, University of Saskatchewan, Saskatoon, SK, S7N 5A2, Canada; E-Mail: kyle.brown@usask.ca

**Keywords:** Andes virus, vaccine, durability, antibody response, hantavirus

## Abstract

Andes virus (ANDV) is highly pathogenic in humans and is the primary etiologic agent of hantavirus cardiopulmonary syndrome (HCPS) in South America. Case-fatality rates are as high as 50% and there are no approved vaccines or specific therapies for infection. Our laboratory has recently developed a replication-competent recombinant vesicular stomatitis virus (VSV)-based vaccine that expressed the glycoproteins of Andes virus in place of the native VSV glycoprotein (G). This vaccine is highly efficacious in the Syrian hamster model of HCPS when given 28 days before challenge with ANDV, or when given around the time of challenge (peri-exposure), and even protects when administered post-exposure. Herein, we sought to test the durability of the immune response to a single dose of this vaccine in Syrian hamsters. This vaccine was efficacious in hamsters challenged intranasally with ANDV 6 months after vaccination (*p* = 0.025), but animals were not significantly protected following 1 year of vaccination (*p* = 0.090). The decrease in protection correlated with a reduction of measurable neutralizing antibody responses, and suggests that a more robust vaccination schedule might be required to provide long-term immunity.

## 1. Introduction

Andes virus (ANDV) is a New World hantavirus (family: *Bunyaviridae*, genus: *Hantavirus*) and is the primary etiologic agent of hantavirus cardiopulmonary syndrome (HCPS) in South America, with case fatality rates of 30%–50%. ANDV is a zoonotic virus hosted by the long-tailed pigmy rice rat (*Oligoryzomys longicaudatus*) and upon transmission to humans, can cause HCPS [1,2]. Human-to-human transmission has also been documented [3,4,5]. Currently, there is no approved vaccine or specific treatment for hantaviruses that cause HCPS, and medical intervention is largely supportive. To date, the only animal model that recapitulates human disease caused by ANDV is the Syrian hamster [6,7]. Our laboratory has recently reported that a recombinant replication-competent recombinant vesicular stomatitis virus (VSV)-based vaccine, engineered to express the glycoprotein complex (GPC) of ANDV in place of the VSV glycoprotein (G), provides complete protection from disease in hamsters [8]. Hamsters were sterilely protected one month after a single dose of the vaccine upon challenge with a consistently lethal dose of ANDV. This vaccine was also efficacious post-exposure, with 90% of hamsters protected when the vaccine was administered one day after inoculation with ANDV.

The mechanism(s) by which this vaccine offers protection is not entirely known, and might differ depending on the time of challenge, as post-exposure protection is unlikely to rely on the generation of neutralizing antibodies, as the VSV-vector expressing an irrelevant glycoprotein also afforded peri-exposure protection against ANDV challenge [8]. Nonetheless, the VSV-based vaccine platform is known to elicit a strong humoral immune response [9]. In the VSV-ANDV system, neutralizing antibodies directed against the GPC are likely important for prophylactic vaccination. Herein, we sought to test the kinetics of the neutralizing response elicited by this vaccine and to test the duration of immunity following a single-dose vaccination to lethal disease caused by ANDV in the hamster model.

## 2. Results and Discussion

**Figure 1:**
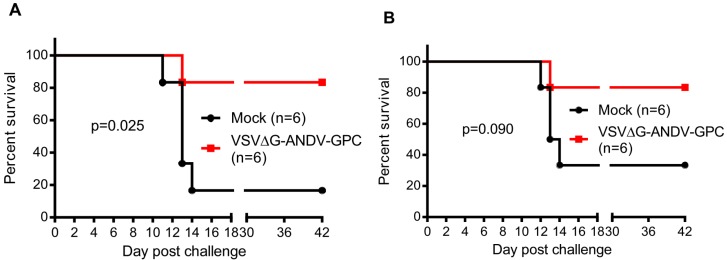
A single dose of a vesicular stomatitis virus (VSV)-based Andes virus (ANDV) vaccine affords significant protection from ANDV disease at 6 months, but not 12 months post-vaccination. Groups of 12 animals were either mock-vaccinated or vaccinated with 10^5^ PFU of VSV∆G-ANDV-GPC i.m., and six animals per group were challenged with 200 focus forming units (FFU) of ANDV i.n. 6 months (**A**) or 12 months (**B**) after vaccination. Animals were monitored for clinical signs of disease and survival for 42 days. Survival was statistically evaluated using a log-rank (Mantel-Cox) test with significance set at 0.050.

Hamsters vaccinated with VSV∆G-ANDV-GPC showed a statistically significant increase (*p* = 0.025) in survival when challenged with a lethal dose of ANDV 6 months after vaccination, with 5 of 6 of the vaccinated hamsters surviving challenge, whereas a single animal of the 6 unvaccinated hamsters survived ANDV challenge (Figure 1A). Conversely, at 12 months after vaccination, the VSV∆G-ANDV-GPC-vaccinated group was not significantly protected (*p* = 0.090) compared to the control group, although the vaccine afforded some level of protection (Figure 1B). At this point, only a single vaccinated hamster developed signs of disease and was euthanized, 2 of the 6 control hamsters survived inoculation, rendering the result at this time point insignificant. It is notable that a single mock vaccinated animal survived at 6 months post-vaccination, and two survived at the 12-month time point. Our laboratory has extensive experience using the Syrian hamster model of HCPS, and experiments are typically, if not always, completed by the time the animals reach 2–3 months of age. We have rarely, if ever, observed a control animal survive this dose and route of ANDV inoculation in control animals, and thus far, all experiments have been performed using the same stock of virus preparation that we used in this study [7,10]. This suggests that age, likely influencing immune status, might play a role in natural protection from disease. Other studies using older hamsters have resulted in more variability in lethality, supporting that older hamsters might be more immune to disease, although this needs to be addressed experimentally and it is difficult to compare studies using different routes of infection, stocks of viruses, and sources of animals [11,12]. A limitation of our study is the relatively small group sizes, which makes it difficult to resolve differences between the potential survival of aged control animals, and discriminating this from the durability of the vaccine. Repeating this study, using larger group sizes as well as additional time points would both lend insight into age-related affects of survival and long-term efficacy of the vaccine.

**Figure 2:**
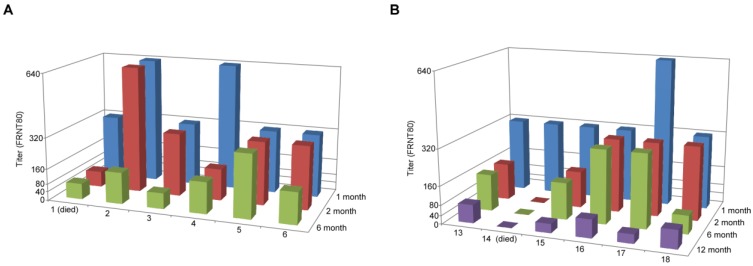
Neutralizing antibodies to ANDV are induced upon vaccination with VSV∆G-ANDV-glycoprotein complex (GPC). Sera were collected from animals at the time points indicated on the *z*-axis and used for an ANDV focus reduction neutralization test 80% (FRNT_80_) as described in the Experimental Section. (**A**) Animals 1–6 were challenged 6 months after vaccination and (**B**) animals 13–18 were challenged with ANDV 12 months post-vaccination and are indicated on the *x*-axis. Animals 1 and 14 developed disease upon ANDV challenge and were euthanized.

To examine the kinetics of the immune response to vaccination over the course of this study, we obtained serum from animals at 1, 2, 6 and 12 months (for the animals remaining after the 6 month challenge experiment) post-vaccination. These sera were tested for their neutralizing activity by performing a FRNT_80_ assay, along with the sera from the unvaccinated animals. None of the mock-vaccinated animals developed measurable neutralizing antibodies (data not shown). All vaccinated animals achieved a titer of at least 320 by 1 month, and three of the 12 animals achieved titers of 640 (Figure 2). In all but two cases, neutralizing titers dropped by 6 months of vaccination, and for a single animal, which developed disease when challenged at 12 months post-vaccination, the titer dropped below 40 (animal #14) (the minimum dilution used for this assay). The hamster that developed disease at 6 months post-vaccination had a neutralizing titer of 80 (animal #1). Two other hamsters in this group also had titers of 80, but did not develop disease, whereas the other three animals had titers of at least 160. For the surviving hamsters that were challenged at 12 months, two had titers of 40 and three had titers of 80. Although all had titers of 80 or less, the observation that they were protected, when the non-protected animal at 6 months had a titer of 80, suggests that the greater age of the animals could contribute to protection. This correlates with the observed increase in survival of the non-vaccinated control animals in the 12-month group, where two survived at this time point, and only one survived at 6 months.

In a previous study, we observed that a single dose of this vaccine provided sterile immunity in most hamsters when challenged 28 days after vaccination [8]. To test whether the surviving animals in this study were sterilely protected from ANDV at 6 and 12 months post-vaccination, we performed an ELISA to detect anti-ANDV-N antibodies (Table 1). All animals that survived challenge, mock vaccinated or vaccinated, developed ANDV-N antibodies, suggesting that sterile immunity was not achieved. This difference could be attributed to the longer time period between vaccination and challenge in this study, and is likely related to our observed decrease in neutralizing antibody titers. We used different routes of inoculation herein, making it difficult to directly compare this study with our previous study. We chose to inoculate hamsters intranasally (with the same infectious dose of virus used previously) to more closely mimic human exposure or transmission, as opposed to the intraperitoneal route, which was previously reported. Mucosal immunity might be more difficult to achieve and confer sterile immunity, which might allow the virus to infect cells of the respiratory tract before being neutralized by the humoral immune response elicited by the vaccine.

Vaccination with VSV-based vaccine vectors has provided potent long-term protection in other systems. Mice vaccinated with VSV expressing the spike protein (S) of severe acute respiratory syndrome virus were protected from lethal challenge 4 months later, following a single dose administration [13]. VSV expressing the HA of influenza virus were protective up to a year after vaccination, although these mice were given a boost of a heterologous VSV-based vaccine prior to challenge [14]. Differences in the platform and animal species used might account for differences in the immune response to this vaccine in the hamster.

**Table 1:** Anti-ANDV N ELISA titers in hamsters that survived i.n. challenge with 200 FFU of ANDV.

Challenge 6 months post-vaccination			Challenge 12 months post-vaccination		
Animal	Vaccine	Titer	Animal	Vaccine	Titer
1	VSV∆G-ANDV-GPC	NA	13	VSV∆G-ANDV-GPC	≥3200
2	VSV∆G-ANDV-GPC	800	14	VSV∆G-ANDV-GPC	NA
3	VSV∆G-ANDV-GPC	1600	15	VSV∆G-ANDV-GPC	≥3200
4	VSV∆G-ANDV-GPC	1600	16	VSV∆G-ANDV-GPC	1600
5	VSV∆G-ANDV-GPC	≥3200	17	VSV∆G-ANDV-GPC	800
6	VSV∆G-ANDV-GPC	≥3200	18	VSV∆G-ANDV-GPC	≥3200
7	Mock	NA	19	Mock	≥3200
8	Mock	NA	20	Mock	≥3200
9	Mock	NA	21	Mock	NA
10	Mock	NA	22	Mock	NA
11	Mock	1600	23	Mock	NA
12	Mock	NA	24	Mock	NA

NA = Animals that did not survive challenge.

## 3. Experimental Section

### 3.1. Hamster Vaccination and Challenge

Female Syrian hamsters 5–6 weeks of age (Harlan Labs, Indianapolis, IN, USA) were administered 10^5^ PFU of VSV∆G-ANDV-GPC by intraperitoneal injection (i.p.), or sterile medium as a control. The VSV∆G-ANDV-GPC was prepared as previously published [8]. Serum samples were obtained at the indicated times post-vaccination by retro-orbital bleeding. The blood was centrifuged at 2,000× *g* for 10 min at room temperature and the serum was removed and frozen for the measurement of neutralizing antibodies. Either 6 or 12 months after vaccination, groups of 12 hamsters (6 mock-vaccinated and 6 vaccinated with VSV∆G-ANDV-GPC) were challenged with ANDV. Two hundred focus forming units (FFU) of ANDV (strain 9717869), which is equivalent to 100LD_50_ when administered i.p., as used in our previous studies, was diluted in 100 μL of sterile medium and was delivered intranasally (i.n.) while the animals were under inhalational isoflurane. Hamsters were monitored daily for signs of disease and were euthanized upon showing signs of severe clinical disease, or at 42 days post challenge, at which time a terminal blood sample was collected to measure serum antibodies.

### 3.2. Andes Virus Neutralization and ELISA

To measure the neutralizing antibody response to vaccination, 2-fold serial dilutions of sera were mixed 1:1 with approximately 100 FFU of ANDV and incubated for 1 h at 37 °C in a humidified chamber. This mixture (200 μL) was then used to inoculate Vero E6 cells (ATCC) for 1 h at 37 °C, 5% CO_2_. The inoculum was then removed and 500 μL of 1.2% carboxymethylcellulose in Modified Eagles Medium (MEM) containing 2.5% FBS was added to the cells and incubated at 37 °C, 5% CO_2_. Seven days later, an immunofocus assay was performed and sera resulting in a greater than 80% reduction in foci were considered positive (FRNT_80_) as previously described [15]. To examine whether hamsters that survived challenge (42 days post inoculation) developed anti-ANDV antibodies, we performed an ELISA to detect antibodies directed against the ANDV nucleocapsid protein (N) as described previously [10].

### 3.2. Statistics

To determine whether vaccination resulted in significant protection from ANDV-induced disease, we compared the survival curves between mock-vaccinated and VSV∆G-ANDV-GPC-vaccinated animals using a log-rank (Mantel-Cox) test with significance set at 0.05. Analysis was performed using Prism software version 6 (GraphPad Software, Inc., La Jolla, CA, USA).

### 3.3. Biosafety and Ethics

All work with ANDV-infected hamsters and potentially infectious material was conducted in the BSL4 facility at the Rocky Mountain Laboratories, Division of Intramural Research, National Institutes of Allergy and Infectious Diseases, National Institutes of Health. Sample removal from the BSL4 was performed according to approved standard operating procedures. This animal experiment was approved by the Institutional Animal Care and Use Committee and performed following the guidelines of the Association for the Assessment and Accreditation of Laboratory Animal Care (AAALAC) by certified staff in an AAALAC-approved facility.

## 4. Conclusions

Vaccination of hamsters with a single dose of a VSV-based ANDV vector provided long-term protection from lethal virus challenge 6 months after administration. We have shown that this vaccine has a high degree of efficacy when administered between 28 days pre-, and 1 day post-challenge, indicating its use in emergency situations, or for laboratory-acquired infections, might prove viable. The decline in neutralizing antibodies at 6 and 12 months indicates that the durability of this vaccine after a single-dose application, at least in hamsters, is questionable. Booster immunizations might improve the durability of this vaccine candidate. Although other disease models have yet to be developed for HCPS-causing hantaviruses, further evaluation of vaccine approaches, specifically measurement of antibody responses, could be warranted in other non-disease animal models.
